# Adjunctive Techniques for Optimizing Percutaneous CT-Guided Cryoablation of Renal Tumours

**DOI:** 10.3390/cancers18060936

**Published:** 2026-03-13

**Authors:** Julien Garnon, Pierre-Alexis Autrusseau, Theo Mayer, Gregory Bertucci, Thomas Fournaise, Julia Weiss

**Affiliations:** 1 Service de Radiologie, Centre de Lutte Contre le Cancer, Institut Strauss, 67200 Strasbourg, France; pierre.autrusseau@gmail.com (P.-A.A.); th.mayer@institut-strauss.fr (T.M.);; 2 Service de Radiologie, Centre Hospitalier de Haguenau, 67500 Haguenau, France; 3 Service de Radiologie, Centre Hospitalier de Polynésie Française, Pirae, 98716 Papeete, France; gregory.bertucci@cht.pf; 4 Service d’Imagerie Interventionnelle, Hôpitaux Universitaires de Strasbourg, 67200 Strasbourg, France; julia-mathilde.weiss@chru-strasbourg.fr

**Keywords:** cryoablation, renal cancer, thermoprotection, balloon occlusion, embolization

## Abstract

Percutaneous computed tomography (CT) -guided cryoablation is an effective curative treatment for renal cell carcinoma. Improvements in treatment efficacy reflect not only the learning curve but also the integration of multiple adjunctive techniques that can be implemented at different stages of the procedure. Tumour targeting can be enhanced by intravenous contrast administration, or by intra-arterial delivery of contrast medium or iodized oil. Fusion imaging is another option to improve tumour delineation by registering intraprocedural CT with prior cross-sectional imaging. Probe placement for difficult-to-access lesions may be facilitated by alternative access routes, while electromagnetic navigation and robotic systems are being developed as alternatives to manual advancement. To mitigate the cold-sink effect and reduce bleeding risk, transarterial techniques such as embolization or temporary arterial occlusion can be added. Finally, thermoprotective manoeuvres are increasingly used to displace adjacent organs, thereby improving the feasibility, safety, and efficacy of renal cryoablation.

## Simple Summary

Percutaneous cryoablation is an effective curative treatment for small renal tumors, with oncologic outcomes comparable to surgery for T1a lesions. However, its efficacy and safety may be reduced in certain situations, particularly for tumors that are poorly visualized, large, or located close to hilar vessels or adjacent organs. Adjunctive techniques can help overcome some of these limitations and improve the feasibility of cryoablation. These notably include transarterial tumor tagging and embolization, transient arterial occlusion, and thermal insulation of surrounding organs. By enhancing tumor visualization and protecting nearby critical structures, these approaches may facilitate treatment of more complex lesions. Adjunctive strategies therefore represent promising tools to expand the indications of percutaneous cryoablation. Nevertheless, larger studies are required to confirm the currently available observational data and to better define their role in clinical practice.

## 1. Introduction

Percutaneous thermal ablation is an effective curative treatment for sporadic, multifocal and hereditary renal cell carcinoma (RCC). Most recent studies show little to no difference in oncological outcomes between percutaneous ablation and partial nephrectomy for T1a tumours [1,2,3,4,5,6]. For T1b tumours, the efficacy of thermal ablation is improving, although it remains inferior to surgery in most reports [4,6,7,8,9]. Despite the growing use of microwave ablation, percutaneous CT-guided cryoablation is typically preferred in high-volume centres [4]. Compared with heat-based modalities (microwave and radiofrequency ablation), cryoablation offers several advantages: (1) real-time estimation of the ablation zone through CT visualization of the ice ball; (2) the ability to treat larger tumours by simultaneously activating multiple cryoprobes, making cryoablation particularly suitable for T1b lesions; (3) better preservation of the urothelium within the collecting system; and (4) lower intra- and post-procedural pain [10,11]. Recent series report complete tumour destruction in >97% of T1a RCCs, a marked improvement compared with early outcomes nearly two decades ago [12,13]. This progress reflects not only operator experience, but also the availability of multiple adjunctive techniques that can be applied throughout the cryoablation workflow to enhance the oncological efficacy and safety of treating tumours, particularly those with challenging size, location, and/or vascularization. The aim of this review is to describe and illustrate these techniques and discuss their indications and limitations.

## 2. Materials and Methods

Tumour residue/recurrence and complications are the main limitations of percutaneous cryoablation for kidney tumours [1,2,3,4,5,6,7,8,9]. In clinical practice, several factors can lead to suboptimal tumour coverage by the ice ball and explain residual or recurrent disease. These include: (1) poor tumour visualization; (2) difficult tumour access; (3) reduction of ice-ball expansion due to the cold-sink effect; (4) tumour size; and (5) proximity of adjacent organs at risk of thermal injury. Complications may result from needle insertion and/or unintended freezing of surrounding vulnerable structures.

We performed a literature review in PubMed and MEDLINE to identify adjuncts to cryoablation that have been described and applied in clinical practice. Search queries used the terms “renal cryoablation” and “kidney cryoablation.” Case reports and studies reporting adjuncts to cryoablation were screened and included in this review if interventions were (1) performed between 2000 and 2025 using percutaneous access and CT guidance and (2) described one or more of the following: (a) optimization of tumour visualization, (b) an approach to the tumour other than the direct transretroperitoneal route, (c) the use of electromagnetic or robotic guidance, (d) the use of additional arterial access, or (e) the use of thermal protective measures.

The main findings are presented in four sections: (1) tumour visualization, (2) probe placement, (3) mitigation of the cold-sink effect and bleeding risk, and (4) thermal protection of adjacent organs.

## 3. Relevant Results

### 3.1. Tumour Visualization

Accurate identification of tumour margins is critical to achieve complete ablation of renal tumours in a single session. Inadequate visualization of tumour size and boundaries may lead to suboptimal probe number and placement, insufficient ice-ball coverage, and ultimately the need for repeat ablation to achieve complete response. Renal tumours exhibit variable morphology and enhancement patterns, and some lesions are irregular and non-ovoid. Beyond appropriate diagnostic work-up, optimal depiction of tumour boundaries on intraprocedural cross-sectional imaging is essential and may be improved by several tools.

#### 3.1.1. Intravenous Contrast Administration

Multiphasic contrast-enhanced CT does not require additional equipment. At the start of the procedure, it facilitates tumour detection, particularly for intraparenchymal and sinusal lesions [14]. For endophytic and central tumours, the benefit of intravenous contrast extends to the probe placement phase, as tumour-to-parenchyma contrast persists during the nephrographic phase for several minutes [15]. In addition, visualization of the collecting system during the excretory (urographic) phase helps (1) avoid traversing calyceal cavities during probe advancement, potentially reducing haematuria and urinary fistula risk, and (2) identify the ureter throughout the procedure, enabling adjunctive thermoprotection and accurate assessment of its distance from the ice ball.

#### 3.1.2. Tumour Tagging Before the Intervention

Several retrospective studies have highlighted the potential value of preprocedural tumour tagging (Table 1) [16,17,18,19]. This approach leverages the hypervascularity of most RCCs to enable intra-arterial delivery of a contrast agent preferentially retained within the tumour. Similar to transarterial therapies for hypervascular liver tumours such as hepatocellular carcinoma, iodized oil (Lipiodol, Guerbet Medical, France) is typically used [16,17,18,19,20]. Tagging is usually performed a few days before cryoablation via femoral arterial access under local anesthesia and fluoroscopic guidance. After renal artery catheterization with a guiding catheter, a microcatheter is advanced into feeding arteries in a selective (segmental) or superselective (subsegmental) position. A small volume (commonly 0.2–0.4 mL) is injected to achieve intratumoural accumulation [16]. Transarterial embolization can be performed during the same session using ethanol, particles, or gelatin sponge [17,18,19].

The main advantage of tagging is improved tumour conspicuity on unenhanced CT at the time of ablation: the hyperdensity of iodized oil creates high tumour-to-parenchyma contrast, facilitating targeting of endophytic lesions [17]. Kajiwara et al. reported 100% technical success of lipiodol tagging in 13 patients, with “very good” lesion identification by two readers on a 5-point subjective scale (1 = invisible; 5 = excellent) [19]. In a series of 99 RCCs, Tsuji et al. reported adequate visualization across histological subtypes, although iodized oil accumulation was significantly lower in chromophobe/oncocytic and papillary RCC compared with clear cell RCC, consistent with their relatively hypovascular nature. In a small subset of six cases, margins remained identifiable despite poor intratumoural retention due to “inverted marking,” i.e., iodized oil deposition surrounding rather than within the tumour [21].

Most authors agree that tagging improves probe placement accuracy and ice-ball coverage assessment without requiring intravenous contrast during ablation [16,17,18,19,21]. However, no comparative study has demonstrated improved local recurrence-free survival relative to non-tagging. A major limitation is the need for an additional arterial procedure, with its inherent (albeit low) access-related risks. Another concern is the potential impact of iodized oil on freezing, given its relatively low thermal conductivity, which may reduce ice-ball size based on ex vivo data [22,23]. Chang et al. showed that 100% iodized oil concentration in a phantom significantly reduced ice-ball size after 9 min compared with normal saline; this effect was not observed at 10% or 40% concentrations [23]. Conversely, a porcine kidney ex vivo model did not demonstrate a negative impact on ice-ball size [24]. Thus, whether tagging compromises cryoablation efficacy remains uncertain, particularly since near-complete intratumoural iodized oil concentration is unlikely in clinical practice [22]. In a retrospective analysis of 18 tumours, Chang et al. reported longer time to reach −100 °C and higher intraprocedural temperatures in the tagging-plus-embolization group than in controls, suggesting a potential adverse effect of iodized oil [25].

Overall, stand-alone tagging (i.e., without concomitant embolization) remains uncommon. It may be useful when CT image quality is expected to be poor (e.g., severe obesity) or when cone-beam CT guidance—typically inferior to diagnostic CT—is used for the ablation procedure.

#### 3.1.3. Arterial Opacification During the Intervention

The development of angio-CT suites enables combined transarterial and percutaneous access within a single intervention. Angio-CT combines an interventional CT scanner and an angiographic cone-beam system in the same unit [26,27]. After patient positioning, the renal artery can be accessed via a transfemoral approach (lateral or supine positioning) or radial approach (prone positioning). A CT acquisition with injection of a small volume of contrast medium (typically 5–10 mL) through a 4F or 5F arterial catheter can provide high renal and tumour enhancement and clear depiction of tumour anatomy in hypervascular lesions [28]. Because each injection uses a limited contrast volume, multiple contrast-enhanced CT acquisitions (up to approximately 20) may be performed before, during, and after cryoprobe insertion.

This is particularly useful when treating endophytic residual disease after a first cryoablation session or when ablating complex, irregular tumours with sinus extension, as it allows sequential verification of each cryoprobe position on contrast-enhanced CT [29]. It is also a valuable option in patients with renal impairment, as the total contrast dose can be kept low while still enabling reliable tumour identification. Arterial opacification can be combined with temporary arterial occlusion (see Section 3.3.2).

In the liver, arterial opacification has been associated with improved local tumour control by enabling more accurate assessment of tumour margins before and during applicator placement [30,31,32]. However, comparable evidence in renal cryoablation remains limited. The main drawback is increased procedural complexity and duration, particularly when radial access is required for prone-position interventions, along with the general risks associated with arterial access.

#### 3.1.4. Fusion Imaging

Current software solutions allow registration of intraprocedural unenhanced CT images with prior contrast-enhanced CT or MRI. Fusion imaging may be performed using rigid registration or, more recently, deformable registration to account for organ deformation related to patient positioning and respiratory motion [33]. It can improve target identification and enhance assessment of probe positioning relative to the tumour compared with native unenhanced images [34]. Evidence has largely been generated in liver ablation, where software-based intraprocedural CT–CT fusion has been associated with improved local tumour control. Fusion imaging can also be applied to renal tumour targeting and may be combined with robotic guidance [35,36,37,38].

A key limitation is the risk of misregistration caused by differences in patient positioning or patient motion during the procedure. In addition, fusion imaging does not account for tissue deformation induced by probe advancement.

### 3.2. Probe Placement

For renal cryoablation, probes are typically placed manually using a direct transretroperitoneal route, advancing through the lumbar and/or intercostal region(s) under intermittent CT guidance to confirm positioning. Several alternative access routes and guidance systems may assist probe placement in challenging cases.

#### 3.2.1. Transhepatic Approach

Upper-pole tumours of the right kidney may be difficult to reach via a direct transretroperitoneal route [39]. Observational studies have reported successful transhepatic access to the right kidney; however, this approach carries a risk of hepatic bleeding [40,41,42]. In a cohort of 23 renal cryoablations performed via transhepatic access, Graif et al. reported two hemorrhages related to the transhepatic route [41].

#### 3.2.2. Artificially Induced Pneumothorax

When the patient is prone, a direct posterior approach to upper-pole renal tumours may be hindered by the intervening lung [39]. Although trans-pulmonary access is technically feasible, it increases the risk of pneumothorax [43]. If a transhepatic route is unsuitable for right-sided lesions, or for left-sided tumours, an artificial pneumothorax can be created to displace the lung away from the probe trajectory [44,45]. This is ideally performed using a blunt-tip needle designed for pleural access and injection of CO_2_ [46]. Because the lung is not traversed, the induced pneumothorax can typically be reabsorbed after probe removal, reducing the risk of delayed pneumothorax.

#### 3.2.3. Guidance Systems

Several guidance systems are available, including electromagnetic navigation and robotic assistance [47]. These tools share the goal of improving targeting accuracy, particularly for difficult-to-access lesions [47]. In a study of 25 upper-pole lesions, accurate cryoprobe placement using an electromagnetic navigation system was reported without transgressing adjacent organs despite oblique trajectories [48]. At present, there is no evidence that guidance systems improve oncological outcomes after renal cryoablation. Their use is therefore largely driven by institutional preference, access to technology, and clinical workflow considerations.

### 3.3. Mitigating the Cold-Sink Effect and Bleeding Risk

The efficacy of thermal ablation can be reduced by the local vascular environment. Hypervascular tumours and/or lesions abutting large vessels are recognized risk factors for incomplete ablation, as blood flow dissipates heat or cold [49,50]. This phenomenon, originally described for heat-based ablation near major vessels and termed the heat-sink effect, also applies to cryoablation as a cold-sink effect and tends to be more pronounced in the central kidney [12,51,52].

The cold-sink effect may predispose to residual tumour in at least two clinical scenarios. First, some RCCs are markedly hypervascular and may exhibit intratumoural dysplastic vessels, arteriovenous shunts, or aneurysms; this is particularly relevant for T1b tumours, where multiple cryoprobes are required to overcome heat dissipation [53,54,55]. This may contribute to the higher risk of incomplete ablation in T1b tumours compared with T1a lesions [13,56,57]. Second, cold-sink effects may occur when tumours are adjacent to sizeable arteries and/or veins, commonly encountered in central tumours with sinus extension. In a large multicentre retrospective study including 1424 tumours, contact with a central renal vessel was identified as a major risk factor for residual unablated tumour after CT-guided percutaneous cryoablation [13].

Bleeding is another important concern given the vascularity of renal parenchyma and tumour tissue. In a multicentre study of 713 renal tumours treated with cryoablation, bleeding occurred in 3.2% of cases and was the most frequent complication [43]. T1b tumours are associated with higher complication rates, particularly bleeding [43,57].

#### 3.3.1. Transarterial Embolization

Transarterial embolization is an effective palliative treatment for symptoms related to advanced RCC (e.g., haematuria and pain) but has limited oncological efficacy as a stand-alone therapy [58]. It can be used preoperatively to reduce blood loss during partial or radical nephrectomy [59]. Several authors have also reported embolization as an adjunct to ablation [60,61,62]. Reducing tumour vascularity before cryoablation may decrease the number of required cryoprobes, improve local control by mitigating cold-sink effects, and reduce post-procedural bleeding risk. These theoretical benefits are most relevant for larger tumours (typically >4 cm).

Embolization is performed via femoral or radial arterial access. After sheath placement, a guiding catheter (typically 4F or 5F) is advanced into the main renal artery under fluoroscopic guidance. Following angiographic identification of tumour feeders, a microcatheter (approximately 1.7–2.8F) is inserted coaxially to selectively catheterize feeders. A range of embolic agents can be used, including liquid agents (ethanol, n-butyl cyanoacrylate glue, and ethylene-vinyl alcohol copolymer), particles (notably polymer microspheres) of variables sizes, coils/microcoils, and gelatin sponge [62,63,64]. The objective is to devascularize the tumour as completely as feasible while minimizing non-target embolization of healthy parenchyma. Selection of embolic material depends on vascular anatomy, tumour vascular pattern, availability, and operator preference. Additional considerations include the degree of CT artefact produced by the embolic agent (which may affect intraprocedural guidance) and the interval between embolization and cryoablation. Longer intervals may necessitate more durable embolization to reduce early revascularization; accordingly, many authors recommend limiting the delay to a few days [60].

An even more streamlined approach is to perform embolization immediately before cryoablation within a single session [61]. Reducing the interval to a few hours permits the use of agents that generate minimal CT artefact (e.g., gelatin sponge or particles) and may require less extensive devascularization, as early revascularization is unlikely. This combined workflow requires both angiographic and CT imaging and can be achieved by transferring the patient between suites or, more efficiently, using cone-beam CT or angio-CT to treat the patient in a single room [26,27].

Most publications support the feasibility and safety of embolization as an adjunct to cryoablation; however, clear clinical benefit remains unproven. In a propensity score-matched retrospective comparison, Gunn et al. found no significant differences in complication rates, renal function change, or haematocrit change between embolization-plus-cryoablation and cryoablation-only groups [65]. A later comparison without propensity matching including T1b and T2a tumours reported that tumours treated with embolization prior to cryoablation were larger and had higher RENAL scores; despite greater complexity, no haemorrhagic adverse events occurred in the combined group, whereas major bleeding was observed in 8.3% of the cryoablation-only group [66]. Renal function decline was similar between groups. Overall, embolization may reduce haemorrhagic complications in larger tumours without additional functional impairment, but there is no evidence that it improves oncological outcomes (Table 2).

Beyond arterial access risks, the main concern with embolization is non-target embolization leading to loss of renal function. In patients with advanced chronic kidney disease, mean loss has been reported at approximately 6 mL/min/1.73 m^2^ [67]. In the general population, renal function decline appears comparable to that observed after cryoablation alone [68]. Embolization is also technically more challenging for central tumours due to complex arterial anatomy and the risk of non-target embolization. Moreover, while embolization may reduce tumour-related cold-sink effects, it cannot safely eliminate cold-sink effects generated by adjacent central arteries or veins, as embolizing those vessels would result in extensive parenchymal necrosis.

In summary, adjunctive embolization may be considered for large hypervascular tumours but should be decided on a case-by-case basis given the lack of prospective comparative evidence.

#### 3.3.2. Transarterial Balloon Occlusion

Temporary occlusion of the renal artery is another strategy to suppress blood flow not only within the tumour but throughout the kidney, including central arteries and veins. Conceptually, this mirrors surgical clamping of the renal pedicle during partial nephrectomy [69]. Theoretical advantages include near-complete suppression of cold-sink effects from both tumour vascularity and adjacent major vessels—an established predictor of incomplete ablation [13]. In an animal study, Nonboe et al. demonstrated that renal artery clamping during freezing increased cryolesion size by approximately 80% and reduced the proportion of viable cells within the intermediate ice-ball zone, without evidence of ischaemic renal injury [70].

Percutaneous temporary occlusion is achieved by inflating a balloon in the main renal artery via transarterial access [28]. This combined technique requires both CT (for probe placement and ice-ball monitoring) and fluoroscopy (for balloon deployment), ideally within an angio-CT suite. Access choice depends on patient positioning: radial access for prone procedures and femoral access for supine or lateral positioning.

After cryoprobes are placed, a 6F guiding sheath is advanced to the ostium of the ipsilateral renal artery. A 0.014-inch guidewire is positioned distally in a segmental branch, and a balloon is advanced either over-the-wire (femoral access) or using a monorail technique (radial access). Balloon diameter is selected to match the arterial lumen. The balloon is inflated in the main renal artery to nominal pressure, and contrast injection through the sheath confirms complete flow arrest. Cryoablation is then performed using a dual-freeze protocol (10 min freeze–10 min passive thaw–10 min freeze). The balloon is typically deflated during the intermediate thaw phase and reinflated before the second freeze to reduce arterial injury risk.

To date, clinical data are limited. In an observational study of 14 central tumours, Autrusseau et al. reported 93% technical success, with one failure of radial access [28]. Median time to achieve vascular access was 30 min and median total procedural duration was 150 min. Primary efficacy (complete ablation after one session) was 93%, with follow-up ranging from 6 to 39 months (median 25 months). One local recurrence at 14 months was successfully retreated using cryoablation with arterial occlusion. Median hospital stay was 2.5 days. Three major complications occurred (two post-ablation syndromes requiring prolonged hospitalization and one asymptomatic pseudoaneurysm treated with embolization). No arterial thrombosis occurred. Mean estimated glomerular filtration rate decline was 16.4 ± 16.2 mL/min/1.73 m^2^.

Although comparative evidence is lacking, temporary arterial occlusion may improve local control for central tumours abutting hilar vessels [13,28,50]. This benefit comes at the cost of longer procedures and potentially higher complication rates and renal function decline. Importantly, ischemia time typically remains below the 25–30 min threshold often cited in surgical literature as a putative limit for ischaemic injury [69]. This technique may offer an alternative to complex partial nephrectomy or total nephrectomy in selected situations (e.g., solitary kidney). Whether it should be reserved for failures of stand-alone cryoablation remains debated and likely depends on institutional expertise.

### 3.4. Thermal Protection of Adjacent Organs

Unintended freezing of adjacent structures can result in serious injury. CT visualization of the ice ball allows real-time assessment of ablation extent relative to nearby organs and improves safety compared with heat-based modalities [10,71]. Nevertheless, complications related to collateral injury still occur. Although cryoablation generally spares the collecting system, the ureteropelvic junction and lumbar ureter remain vulnerable and may develop stenosis and upstream dilatation after freezing injury [72,73,74,75,76,77]. Peripheral at-risk structures include the bowel (duodenum, small bowel, colon), with risk of perforation; abdominal wall nerves (ilioinguinal, iliohypogastric, genitofemoral), with risk of pain or abdominal wall pseudohernia; the pancreas, with pancreatitis risk; and the adrenal gland, with risk of hypertensive crisis or adrenal insufficiency [78,79,80,81,82,83].

Historically, a minimal safety distance of 5–10 mm between the tumour and adjacent organs was considered necessary for percutaneous cryoablation, and anterior tumours were often treated laparoscopically to allow surgical mobilization [84]. Adjunctive thermoprotective techniques enable percutaneous displacement of adjacent organs, thereby expanding indications for percutaneous cryoablation and rendering most lesions accessible [85,86,87,88,89]. Beyond safety, thermoprotection may improve efficacy by allowing larger ice-ball margins, which ideally extend >5 mm beyond the tumour [87,90,91]. For T1b tumours, an ice-ball margin >8 mm has been associated with reduced residual/recurrent disease risk, implying that organs within 10 mm of the tumour edge should ideally be displaced [90].

Thermoprotection techniques include external compression, probe torquing, hydrodissection, carbodissection, and balloon interposition [46]. External manual displacement has largely been superseded by other methods [92]. Probe torquing can lever the kidney using ice-fixed probes but usually allows limited movement and has modest clinical utility [93,94]. Hydrodissection involves inserting a small-calibre needle (15–22 G) between the tumour and the adjacent structure and injecting saline or 5% dextrose solution, typically 250–500 mL, to widen the separation. Adding a small amount of contrast to the injected fluid is recommended to monitor distribution, which differs according to the anatomical space in which it is injected [95]. Infusion can be continued during freezing to maintain separation or provide active thawing if needed. Endoluminal fluid instillation (pyeloperfusion) can similarly protect the ureteropelvic junction and ureter. Carbodissection uses CO_2_ via a dedicated system; CO_2_ is preferred over air due to its solubility and reduced risk of embolism. CO_2_ is readily visible on CT and provides insulation due to low thermal conductivity; however, it may distribute preferentially to non-dependent spaces and may be rapidly absorbed, potentially reducing mid-procedure protection [46]. Balloon interposition is typically reserved for failure of hydro- and/or carbodissection and involves placing and inflating an angioplasty or oesophageal balloon between the tumour and the organ at risk. Although balloon interposition can achieve >10 mm separation, it is technically more demanding and balloons may migrate, sometimes requiring multiple devices [96].

#### 3.4.1. Thermoprotection for Central Tumours

If the ice ball is not expected to extend beyond the calyceal cavities, cryoablation can often be performed safely without additional protection. Animal studies have shown urothelial viability despite haemorrhagic changes, explaining why contact with the collecting system may cause self-limited haematuria without fistula or focal calyceal stenosis—an important distinction from heat-based modalities. Clinical series have reported safe cryoablation of renal sinus tumours without pyeloperfusion, with bleeding complications but no urinary injury [97,98]. By contrast, freezing the ureteropelvic junction and/or lumbar ureter increases the risk of fibrotic change and stenosis, warranting caution for inferior pole tumours or lesions extending deeply into the sinus near the junction [74,75,76,77].

Protection strategies include pyeloperfusion (retrograde or antegrade), ureteral stenting, hydrodissection, carbodissection, and balloon interposition (Table 3). Retrograde pyeloperfusion requires cystoscopic catheter placement by a urologist, usually on the procedure day, with continuous saline infusion during freezing; its main limitation is logistical complexity [99]. Antegrade pyeloperfusion resembles nephrostomy placement and can be performed in the interventional suite; a guidewire is placed into the collecting system and a 4–5F catheter or sheath is advanced to allow infusion. Using a ≥5F catheter enables retention of the guidewire during infusion, which can facilitate ureteral displacement or confirm that the wire is not frozen within the ice ball by gentle manipulation [100]. Limitations include the challenge of targeting a non-dilated system and the bleeding risk of a transparenchymal puncture. In a series of 67 tumours treated with cryoablation and pyeloperfusion where the ice ball contacted the ureter, urinary complications still occurred, indicating that pyeloperfusion does not completely eliminate risk [101]. Some authors therefore advocate JJ stent placement after pyeloperfusion.

Alternatively, a JJ ureteral stent may be placed a few days before cryoablation and left in situ for at least two months, allowing intentional freezing of the ureteropelvic junction/lumbar ureter while using the stent as a scaffold to prevent tight delayed strictures [102]. This approach avoids limiting ice-ball expansion and may therefore optimize oncological margins, but early stent-related complications may occur. Hydrodissection has also been used to protect the ureteropelvic junction and/or ureter without pyeloperfusion by placing one to three needles between the tumour and the collecting system and performing continuous saline injection during freezing; favourable safety outcomes have been reported [103]. CO_2_ and balloon interposition have been used similarly, though these techniques require a feasible access plane between tumour and ureter, which is not always present.

Oncological outcomes in this context are variable. Some focused series report favourable local control approaching typical renal cryoablation outcomes, whereas larger cohorts identify ureter proximity and/or the use of pyeloperfusion as predictors of incomplete ablation or tumour progression [104,105]. These lesions are often central and may be affected by cold-sink effects, which may partly explain poorer outcomes.

#### 3.4.2. Thermoprotection for Peripheral Tumours

Peripheral renal tumours may contact multiple adjacent structures, including the liver, gallbladder, colon, small bowel, duodenum, abdominal wall muscles and nerves on the right, and the spleen, pancreas, colon, small bowel, and abdominal wall structures on the left. In one large cohort, the colon was the most common organ requiring protection [106]. Hydrodissection and carbodissection are generally the simplest and least expensive protection methods. Their effectiveness depends on the retroperitoneal space targeted and on the injected volume, noting that CO_2_ distribution is more gravity-dependent and may be absorbed more rapidly than fluid. Switching between hydro- and carbodissection is feasible when initial separation is inadequate. The retromesenteric space may be more effective than the perirenal space for achieving organ separation, although intercompartmental communication may occur [95]. In most cases, >1 cm separation can be achieved, allowing adequate oncological margins with low complication risk.

Thermoprotection for peripheral tumours is highly effective for preventing collateral injury. Retrospective studies have reported high rates of successful organ separation and very low rates of at-risk organ injury, even for tumours in direct contact with adjacent structures [106,107]. As such, thermoprotective measures should be considered essential adjuncts in many cases and have contributed to the shift towards percutaneous cryoablation as a credible alternative to partial nephrectomy, including for anterior tumours.

### 3.5. Clinical Cases

Clinical cases illustrating the different adjunctive techniques are presented as Supplementary Material.

Case1 (Figure S1) describes the benefit of intravenous contrast administration and hydrodissection during cryoablation of an endophytic T1a RCC.

Case2 (Figure S2) describes the benefit of hydrodissection to treat an anterior T1a RCC that would not be treatable with cryoablation without organ displacement.

Case3 (Figure S3) presents the benefit of intra-arterial opacification to identify a small residual RCC following initial cryoablation of a T1b RCC. The treatment was combined with pyeloperfusion to decrease the risk of pyeloureteral cryoinjury.

Case4 (Figure S4) presents the technique of intravascular arterial occlusion combined with pyeloperfusion for the treatment of a central RCC extending along the hilar vessels.

## 4. Conclusions and Future Directions

The different adjuncts to cryoablation present variable levels of complexity and interest. While identification of the index tumour and displacement of surrounding organs may facilitate the treatment in a substantial number of cases, other options such as transarterial approaches may only be used for specific situations. The goal is ultimately to increase the size of the safety margins without compromising safety, thereby leading to better oncological outcomes. There is still a need for more evidence supporting translation to better oncological outcomes.

## Figures and Tables

**Table 1:** Summary of publications focusing on tumour tagging prior to cryoablation.

Reference	No of Patients	Technical Success of Tagging	Main Findings of the Study	Conclusion of the Study
[16]	31 patients in the tagging group/ 11 in the non-tagging group (no matching)	not specified	• no progressive disease in the tagging group • 4 progressive diseases in the non-tagging group • 0% rate of hemorrhagic complication in the tagging group • 18% rate of hemorrhagic complication in the non-tagging group	Although further studies involving more patients are needed in order to evaluate long-term results, cryoablation therapy appears to be a useful treatment option for SRM. Preoperative marking with lipiodol was helpful for improving complication and survival rates with cryoablation.
[17]	17 patients/17 tumours	94%	• one local recurrence at a mean follow-up of 15.4 months • glomerular filtration decline of 8% (statistically significant) • no complication	TAE using a mixture of absolute ethanol and iodized oil to improve visualization of endophytic renal masses facilitated tumour localization on unenhanced CT, permitting depiction of the tumour edge as well as a safe margin for ablation during CT-guided PCA, with an acceptable decline in renal function.
[18]	29 patients/42 tumours	97.5%	• one local tumour progression at a mean follow-up of 13.9 months • one complication (hypertensive crisis) • no significant decline of glomerular function	Lipiodol marking and embolization (LME) using iodized oil and gelatin particles to improve visualization of the RCC facilitated tumour localization on unenhanced CT images. Percutaneous cryoablation after LME might be a safe and effective for treatment in patients with RCC.
[19]	10 patients/13 tumours	100%	• median visualization score 4/5 (very good) at the time of cryoablation • mean CT density = 437 HU at the time of cryoablation • no recurrence at a median follow-up of 11.5 months • no complication	As the transarterial infusion of iodized oil and gelatin particles improved RCC visualization on CT, its delivery before CT-guided PCA may improve its safety and success rate in patients with RCC.
[21]	99 patients/99 tumours	not specified	• mean density was higher in clear cell RCC than in chromophobe/oncocytoma RCC and papillary RCC • even with poor intratumour iodized oil retention, sufficient visualization was obtained due to “inverted marking”	Regardless of the tumour subtypes, ethiodized oil marking aids in visualizing the boundary between the tumour and parenchyma on non-contrast CT in PCA.

**Table 2:** Summary of publications studying the addition of embolization to cryoablation for renal tumours.

Reference	Indication for Embolization	No of Patients	Main Findings of the Study	Conclusion of the Study
[60]	Tumour > 3 cm	46	• Primary and secondary efficacy rates for T1a tumours: 93% and 100% • Primary and secondary efficacy rates for T1b tumours: 81% and 87% • 2 severe post-procedural hemorrhages	Sequential transarterial embolization and percutaneous cryoablation is a technically feasible and effective treatment option with a low severe adverse event rate for renal masses > 3 cm.
[61]	Tumour > 3 cm	11	• Primary and secondary local tumour control rates at a mean follow-up of 5.2 years: 82% and 100% • No change in renal function	A combined treatment of selective transarterial embolization and percutaneous ablation of large centrally located RCC (>3 cm) is safe and feasible and can achieve excellent oncological long-term results. Larger prospective studies are needed.
[65]	not specified	9 patients compared to 18 patients (cryoablation without embolization) using propensity score matching	• No difference in complication rates • No difference in change in renal function • No difference in change in hematocrit	TAE of RCC prior to PCA is safe and technically feasible; however, no objective benefits over PCA alone were identified by propensity score matching analysis. Due to small sample size and limitations of the study, no definite conclusions should be drawn. Larger, prospective studies of this therapeutic approach are warranted.
[66]	T1b and T2a tumours	13 patients compared to 35 patients (cryoablation without embolization) without propensity score matching	• Larger diameter and higher RENAL score in the embolization + cryoablation group • No difference in local tumour control • Higher hemorrhagic event in the group without embolization	TAE + PCA in patients with T1b or T2 RCC is technically feasible without significant added detriment to renal function. This combined approach may help to reduce hemorrhagic AEs but larger patient cohorts are needed.
[67]	Tumour in non-dialysis patients with stage 4 or 5 chronic kidney disease	9	• No local tumour progression • In 2 patients, hemodialysis was initiated at 3 and 19 months after cryoablation	Cryoablation combined with TAE for RCC in non-dialysis patients with stage 4 or 5 CKD was effective and safe, with an acceptable impact on renal function.

**Table 3:** Summary of publications focusing on protection of the pyeloureteral junction/ureter.

Reference	No of Patients	Technique for Protection of the Pyeloureteral Junction/Ureter	Main Findings of the Study	Conclusion of the Study
[99]	6	Antegrade pyeloperfusion (*n* = 3) Retrograde pyeloperfusion (*n* = 3)	• No ureteral stricture • No tumour recurrence at a median follow-up of 37.3 months	This study demonstrates the feasibility of antegrade and retrograde warm saline perfusion for ureteral preservation during cryoablation, without compromising oncologic outcomes.
[101]	67	Retrograde pyeloperfusion	• Technical success of pyeloperfusion: 99% • Urinary complication: urine leak = 3, transient urinary obstruction = 2, ureteropelvic junction stricture = 1 • Local recurrence-free survival of 92% at 3 years	Retrograde pyeloperfusion of the renal collecting system is a relatively safe and efficacious option for ureteral protection during renal tumour cryoablation. This adjunctive procedure should be considered for patients in whom cryoablation of a renal mass could potentially involve the ureter.
[102]	61	Pre-operative ureteral stenting	• Major complication rate: 10% (including urinary infection, stent displacement, kidney obstruction) • No association between complications and whether the ice ball was touching the ureter	This multicenter cohort study found a relatively high rate of postoperative complications in patients receiving a ureteral stent before PCA. However, no complications resulted in a chronic outflow obstruction. The number of high-complexity tumours could explain the high rate of complications.
[103]	20	Hydrodissection	• Mean of 1.7 ± 0.6 spinal needles to complete hydrodissection • Mean time to complete hydrodissection of 18.6 ± 13.4 min • Primary and secondary efficacy rate: 90 and 95% • Two local recurrences at a mean follow-up of 23.1 months • No ureteral stricture	Hydrodissection of the PUJ/ureter to prevent thermal injury during cryoablation is an effective technique and does not seem to compromise the efficacy of ablation at short or mid-term follow-up.

## Data Availability

No new data were created or analyzed in this study. Data sharing is not applicable to this article.

## Supplementary Materials

The following supporting information can be downloaded at https://www.mdpi.com/article/10.3390/cancers18060936/s1: Figure S1: Cryoablation of an endophytic renal cell carcinoma; Figure S2: Cryoablation of an anterior renal cell carcinoma; Figure S3: Cryoablation of an endophytic renal cell carcinoma; Figure S4: Cryoablation of a renal cell carcinoma with sinusal extension.

## Abbreviations

The following abbreviations are used in this manuscript:

CT	Computed tomography
RCC	Renal cell carcinoma
