# Supplementary material for: Adjunctive Techniques for Optimizing Percutaneous CT-Guided Cryoablation of Renal Tumours
Source: Cancers (Basel). 2026 Mar 13;18(6):936. doi: 10.3390/cancers18060936 (PMC13024733; doi:10.3390/cancers18060936)
Supplement: Supplementary file 1 [file cancers-18-00936-s001.zip › cancers-4132207-supplementary.pdf]

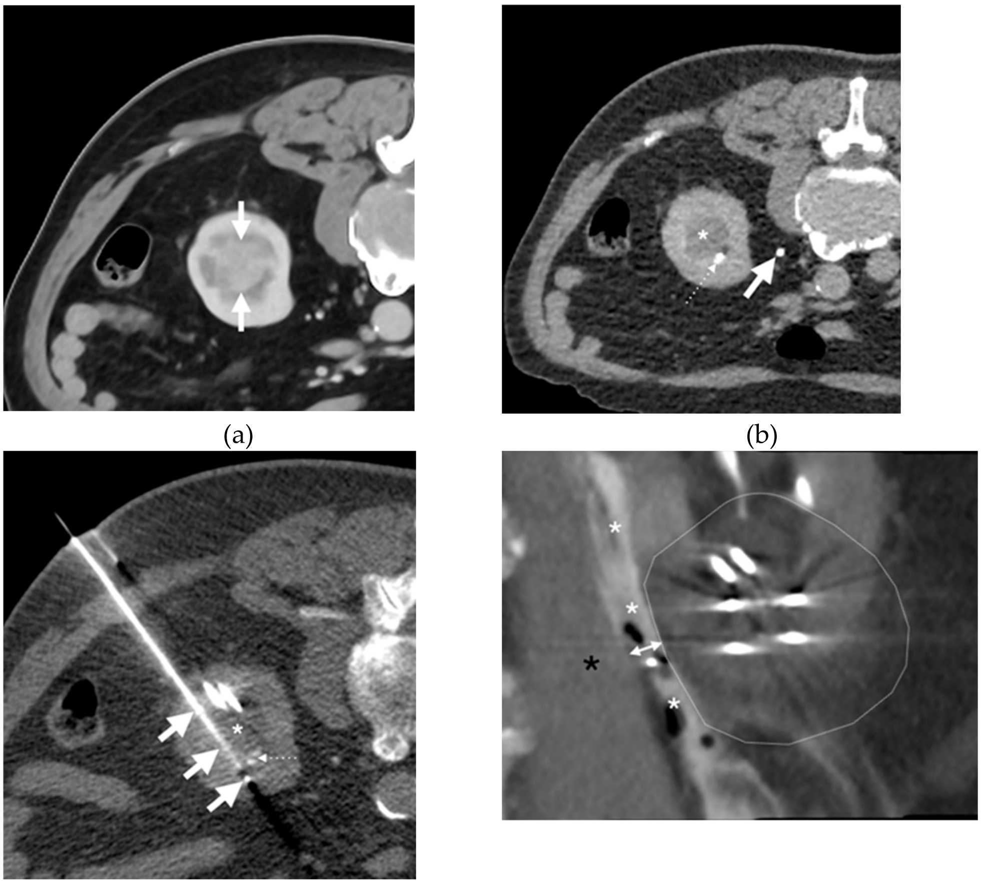

Figure S1. Cryoablation of an endophytic renal cell carcinoma. (**a**) contrast-enhanced planning CT-scan depicts clearly the tumor (arrows); (**b**) CT-scan 15 minutes after injection before insertion of the cryoprobes shows the tumor (asterisk) as a hypodense structure. The inferior caliceal cavity filled with contrast (dashed arrow) is clearly visible, as well as the ureter; (**c**) 60 min after injection, the 5th cryoprobe (arrows) is inserted. The tumor (asterisk) is still visible and the tip of the cryoprobe is advanced without transfixing the caliceal cavity (dashed arrow) thanks to its clear identification on CT images; (**d**) coronal CT-scan at the end of cryoablation shows a large iceball (arrows) covering the inferior pole of the kidney. Hydrodissection (white asterisks) separates (double arrow) the iceball from the psoas muscle (black asterisk).

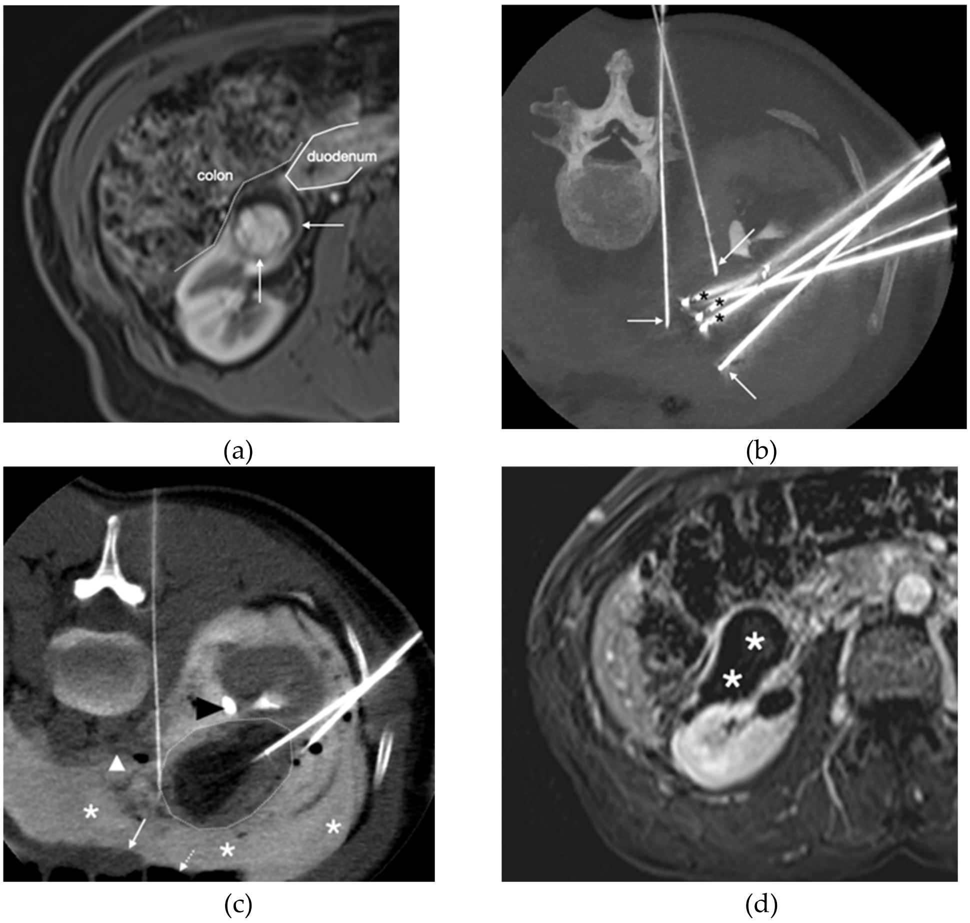

Figure S2. Cryoablation of an anterior renal cell carcinoma located. (**a**) axial contrast-enhanced MRI shows a tumor (arrows) with both solid and cystic components abutting the duodenum and the colon ; (**b**) Three needles (arrows) were inserted in the retroperitoneal space to perform hydrodissection for protection of the digestive structures and of the peyloureteral junction. Four cryoprobes (asterisks) were positioned into the tumor; (**c**) following injection of 1.5 L of saline mixed with contrast, there is extensive hydrodissection of the retroperitoneum (asterisks) that displace the duodenum (white arrowhead), the small bowel (arrow) and the colon (dashed arrow) away from the iceball (dashed circle). The pyeolureteral junction (black asterisk) is identified thanks to contrast opacification. Its nearness to the iceball is well appreciated on CT images; (**d**) 6 months follow-up MRI shows complete necrosis (asterisks) of the mass. There was no injury to the bowel or the pyelo-ureteral junction.

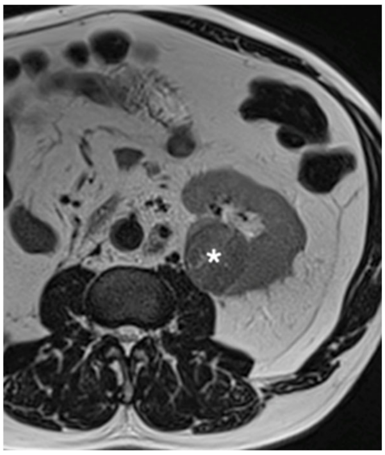
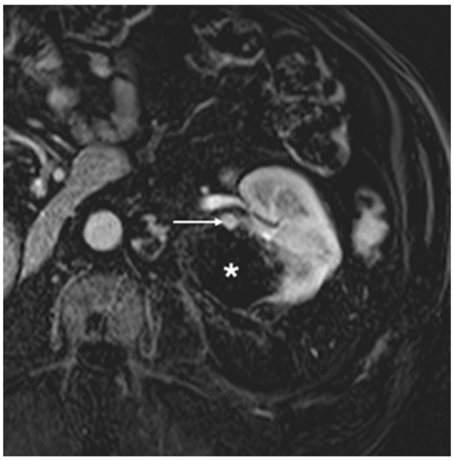

(a) (b)

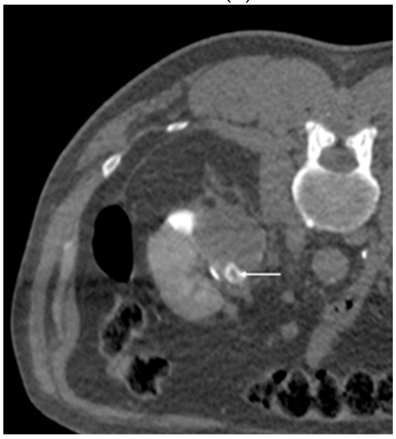
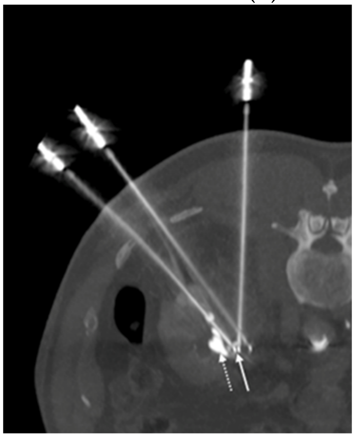

(c) (d)

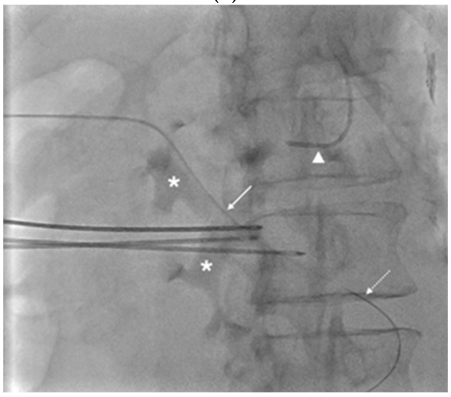
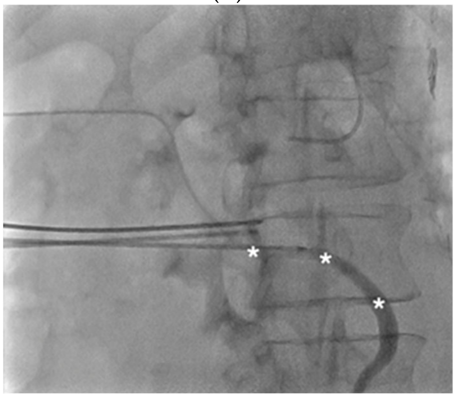

(e) (f)

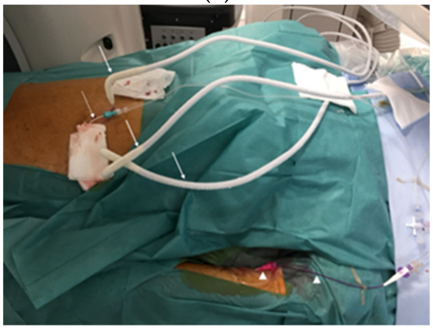
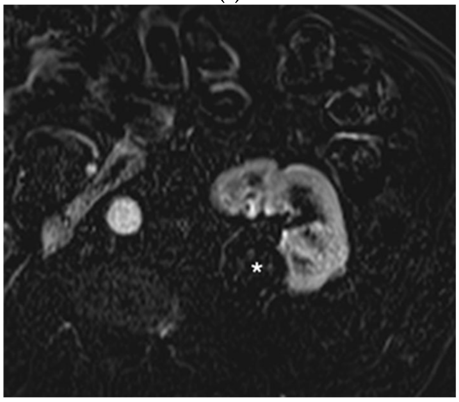

(g) (h)

Figure S3. Cryoablation of an endophytic renal cell carcinoma. (**a**) axial T2 MRI shows a tumor (asterisk) located on the posterior part of the left kidney (single kidney); (**b**) axial contrast-enhanced 3 months after the cryoablation procedure (not shown) shows a large necrotic area (asterisk) with a residual nodule (arrow) located centrally; (**c**) axial CT scan with patient in prone position. The injection is performed with 10 ml through a catheter located in the left renal artery (not shown). The residual nodule (arrow) is perfectly visible; (**d**) another contrast-enhanced CT-scan is performed to assess that the 3 cryoprobes encompass the residual nodule (arrow). Note the proximity of the pyeloureteral junction(dashed arrow); (**e**) a sheath (arrow) is advanced over the wire (dashed arrow) into the collecting system (asterisks). Note the catheter located into the renal artery (arrowhead); (**f**) injection of contrasted saline through the sheath confirms proper diffusion of the fluid into the pyeloureteral junction and the ureter (asterisks); (**g**) external view of the interventional field with the patient prone (head is towards the left side): the 3 cryoprobes (arrows), the sheath for pyeloperfusion (dashed arrow) and the arterial catheter through radial access (arrowheads) are visible; (**h**) axial contrast-enhanced 3 months after the 2nd cryoablation procedure shows complete destruction of the residual nodule.

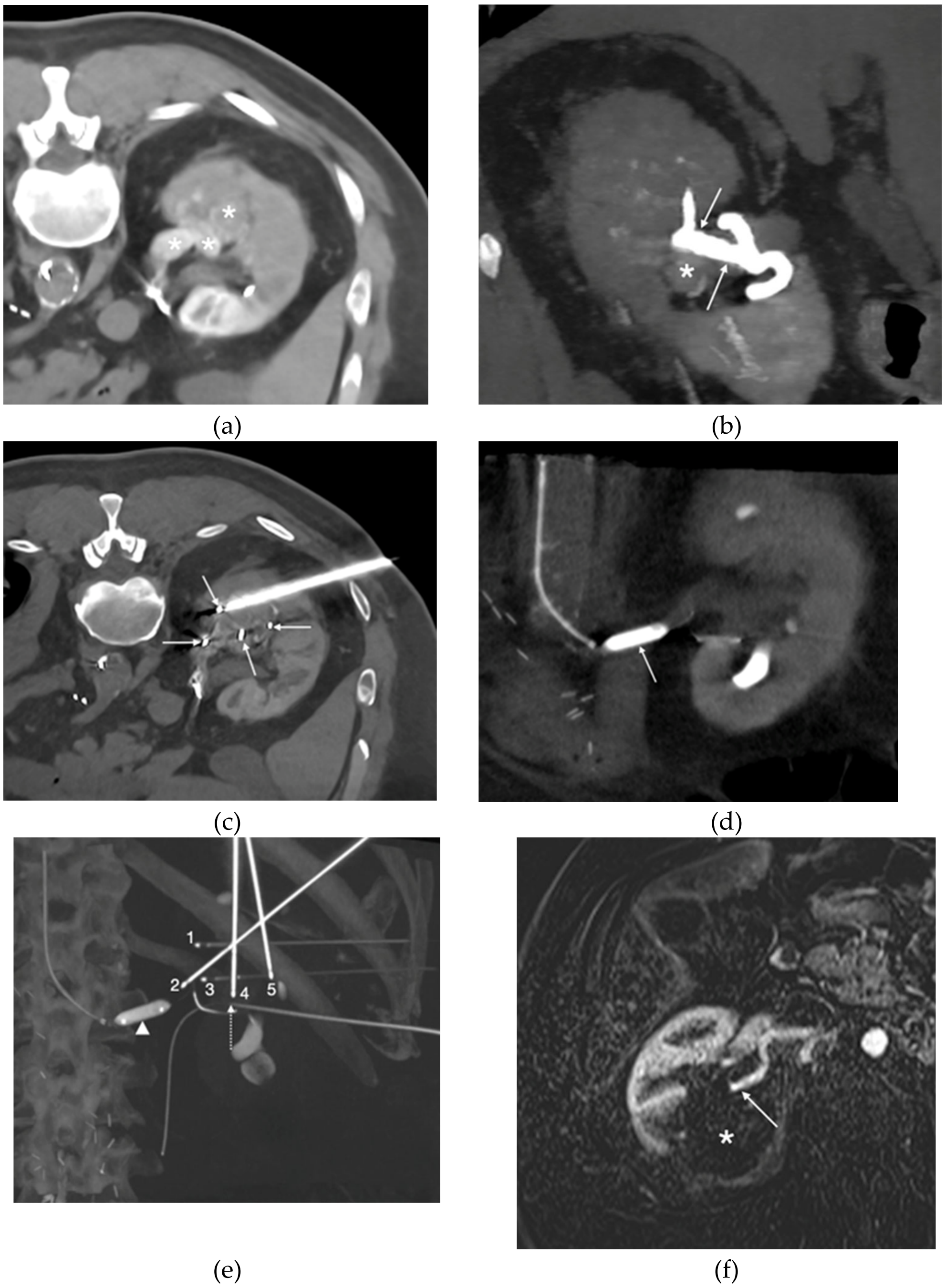

Figure S4. Cryoablation of a renal cell carcinoma with sinusal extension. (**a**) contrast-enhanced CT-scan with injection through a catheter into the renal artery demonstrates an ill-defined tumor (asterisks) extending centrally into the renal sinus; (**b**) sagittal CT-scan shows that the tumor (asterisk) has a large contact with a segmental artery (arrows); (**c**) contrast-enhanced CT-scan with injection through the catheter confirms that the cryoprobes (arrows) are well positioned into the lesion; (**d**) occlusion of the renal artery with a balloon (arrow) advanced via a radial access; (**e**) overview of the intervention with 5 cryoprobes, arterial occlusion with a balloon (arrowhead) and pyeloperfusion via a sheath inserted into the collecting system (dashed arrow); (**f**) axial contrast-enhanced 3 months after cryoablation shows complete destruction of the lesion (asterisk) including next to the segmental artery (arrow).
